# Supplementary figures and images for: Controlled and Synchronised Vascular Regeneration upon the Implantation of Iloprost- and Cationic Amphiphilic Drugs-Conjugated Tissue-Engineered Vascular Grafts into the Ovine Carotid Artery: A Proteomics-Empowered Study
Source: Polymers (Basel). 2022 Nov 26;14(23):5149. doi: 10.3390/polym14235149 (PMC9736446; doi:10.3390/polym14235149)

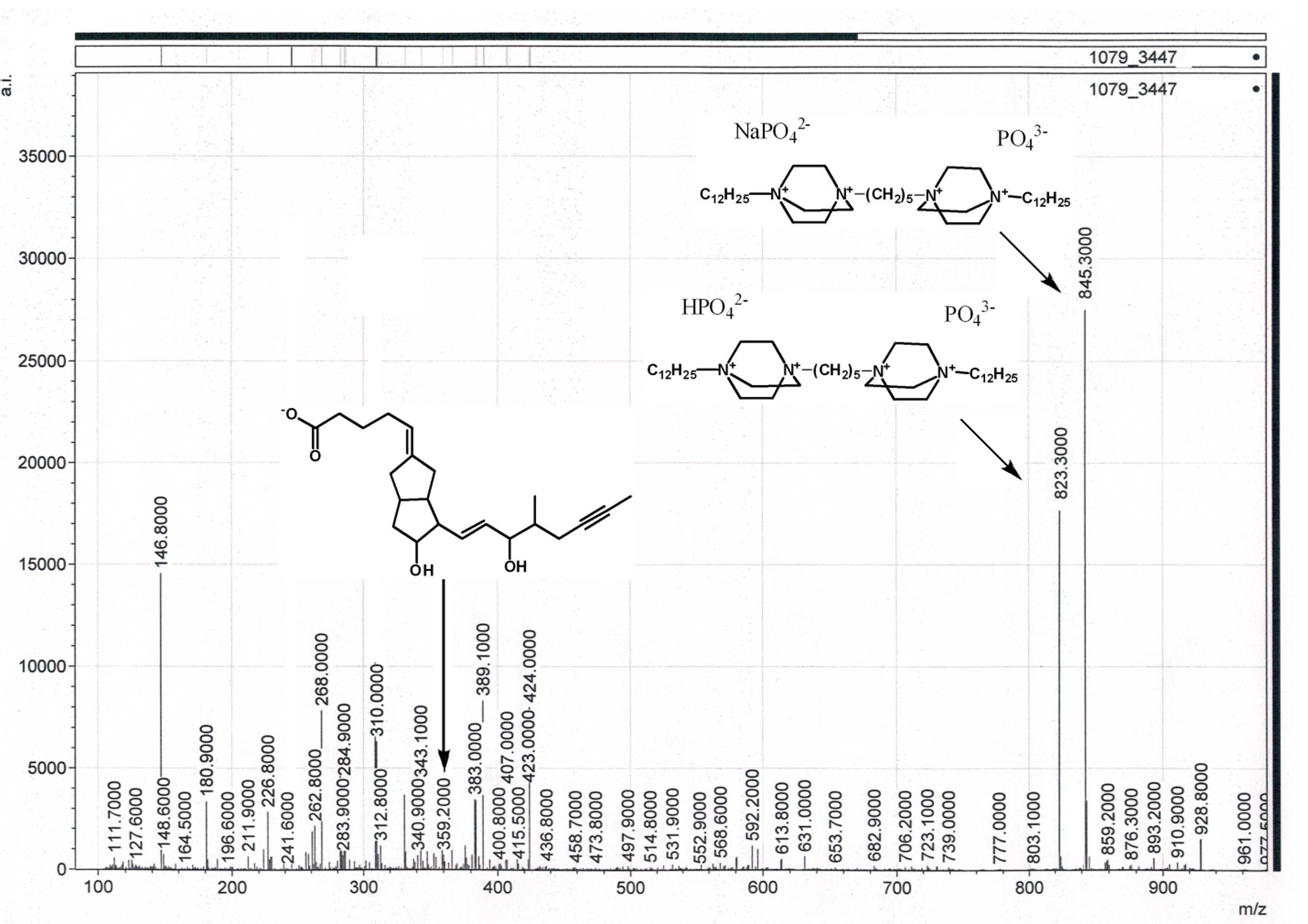

Supplement: Supplementary file 1 [file polymers-14-05149-s001.zip › polymers-2017391-supplementary.tif]
